# Mapping the mismatch between building and population growth: A global study of 1,700 cities

**DOI:** 10.1016/j.isci.2025.113289

**Published:** 2025-08-05

**Authors:** Siwei Lou, Yu Huang, Yukai Zou, Dawei Xia

**Affiliations:** 1 School of Civil Engineering and Transportation, Guangzhou University, 230 Guangzhou Higher Education Mega Center West Outer Ring Road, Panyu District, Guangzhou 510006, China; 2 School of Architecture and Urban Planning, Guangzhou University, 230 Guangzhou Higher Education Mega Center West Outer Ring Road, Panyu District, Guangzhou 510006, China

**Keywords:** Geography, Regional geography, Urban planning

## Abstract

The proportionality between urban building expansion and population growth is a key indicator of sustainable development, yet its global patterns and drivers remain underexplored. This study investigates the discrepancies between building volume and population growth rates (Δ*R*) across 1,744 cities in 12 major global economies from 2000 to 2020 using satellite-based building volume and population databases. Results reveal significant regional disparities: cities in East and Southeast Asia tend to construct buildings at rates exceeding population growth, while many cities in Europe and North America show conservative building developments with Δ*R* close to or lower than zero. Socioeconomic factors such as gross domestic product (GDP), population size, and spatial clustering around the regional economic centers further explain these patterns, particularly in economies of the Global South. These findings emphasize the need for region-specific approaches and strategies to achieve sustainable urban development.

## Highlights

• Mismatches in building and population growth were examined in 1,744 cities globally
• The mismatch indicator detects the urban underbuilding or overbuilding trends
• A Global North-South divide in the mismatch patterns was identified and analyzed
• Overbuilding in smaller cities can be triggered by nearby economic centers

## Graphical abstract

**Figure:**
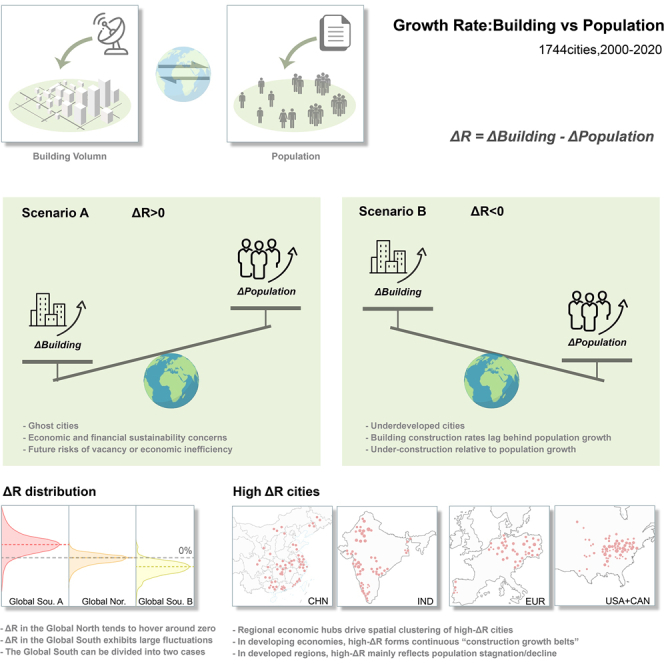

## Introduction

The rapid development of urban areas and their associated infrastructure has profoundly impacted the environment and society. In modern urban constructions, the expansion of the built-up regions has far-reaching impacts on urban density,^1^ urban form,^2^ land use efficiency,^3^^,^^4^ and the preservation of arable land and ecosystems.^5^^,^^6^ However, the uncontrolled urban growth often leads to unplanned and excessive development,^7^^,^^8^ rapid land use changes,^9^^,^^10^ and a host of environmental consequences such as urban heat islands,^11^ air pollution,^12^^,^^13^ urban flooding,^14^ intensive energy consumption,^15^ and social inequalities in living conditions and access to services.^11^^,^^16^

Cities strive to meet the increasing demands of their population for residential and social spaces while maintaining affordability and fostering cooperation among their residents.^17^ As the population grows, the expansion of urban areas and the development of building spaces become inevitable trends, concentrating industries and people within and around cities.^18^^,^^19^^,^^20^ While population growth is often highlighted as the primary driving force behind urban expansion and the relevant building constructions,^21^^,^^22^ the process is complex and can be influenced by various factors. These factors include local policies,^23^ governance effectiveness,^22^ socioeconomic conditions,^24^ and competitive pressures among the surrounding cities to boost economic growth.^25^ Consequently, the cities can become overdeveloped or underdeveloped when urban growth exceeds or fails to match their population status and future trends.^26^ For instance, regions with excessive building construction speeds can exceed the demands of the local population,^27^^,^^28^ which are sometimes labeled as “ghost cities” by the media, raising concerns about the economic and financial sustainability of the city’s development modes and the relevant industries.^29^ Additionally, the mismatch can be caused in cities with a declining population due to factors such as city decentralization, population aging, and emigration.^30^^,^^31^

Therefore, instead of criticizing the environmental and social consequences of urban expansion, it can be more reasonable to evaluate the status of the cities’ development speeds by referring to the population sizes and changing trends. Although several case studies have analyzed the rationality of city expansions from the perspective of building occupancy and vacancy rate, these investigations are often constrained by limited data availability and privacy concerns. Such studies frequently rely on indirect indicators such as nighttime lighting^32^ or micro-scale indicators like household power consumption,^33^ which introduce uncertainty and limit the generalizability of their findings.

However, existing research on urban expansion exhibits limitations. When quantifying the city expansion, most current studies focus on the change in the spatial area and topology of the developed regions across the land surface,^34^^,^^35^^,^^36^^,^^37^^,^^38^ probably because of the good accessibility of the “two-dimensional” city region data. The building volume in three dimensions and its change rates, however, have been less frequently examined^39^^,^^40^ despite their significant environmental^41^^,^^42^^,^^43^ and socioeconomic impacts.^44^^,^^45^ Though the two-dimensional analyses can effectively evaluate land use features for general studies, they cannot fully capture the urban scale, building stock, and population capacity in detail. For instance, redeveloping low-density areas like urban villages^46^^,^^47^ or brownfields can substantially increase building volume and population capacity. Yet, such changes are often not fully reflected in the conventional surface-based land use metrics. Consequently, the demand-and-supply status of the building area for residential, commercial, and public activities, as well as the socioeconomic consequences of building constructions and city (re)developments cannot be fully depicted without considering three-dimensional building data.

The recent studies, though considering the building stocks or volumes, have mainly focused on individual cities^48^^,^^49^ or specific regions,^50^^,^^51^ with few addressing urban expansion at a global scale. Research at the country and international levels is scarce, with only a few global-scale building volume databases covering specific years and even fewer offering long-term data.^52^ Additionally, most current studies focus on several cities in China, Europe, or North America,^53^ with limited comparisons on factors such as urban expansion speeds and land use efficiency across cities or regions with different urbanization and sociodemographic status. As a result, while localized or regional studies provide valuable insights into urban expansion characteristics, especially in well-urbanized areas, the lack of comparative analysis limits the generalizability of findings, particularly in areas with limited urban development levels at present but significant potential in the future.

In summary, although existing studies have identified some key features of city expansions, significant uncertainties remain regarding the drivers of disproportionate urban expansion and population growth. If and how do the geographic, socioeconomic, and demographic factors influence these disproportionate growth of a city? Are there any similarities or differences in the disproportion within and across countries or regions? What spatial or quantitative characteristics distinguish areas where urban development outpaces or lags behind population growth? With global urbanization accelerating and population growth continuing, there is an urgent need to address these questions, evaluate city growth’s rationality according to the stock of buildings, and understand the influential mechanisms. Therefore, this study compares the change rates in building volume and population size across 1,744 cities in major global economies that are either urbanized or under intensive urbanization process over 20 years. By focusing on the differences in building volume and population growth rates, we assess whether cities are developing sustainably, identify potential risks of overdevelopment or underdevelopment, and explore correlations between urban growth patterns and key geospatial and socioeconomic drivers. The socioeconomic and geospatial features of the cities with disproportionate growth and their implications for future urban developments are discussed.

## Results

### Change rates of building and population

**Figure 1:**
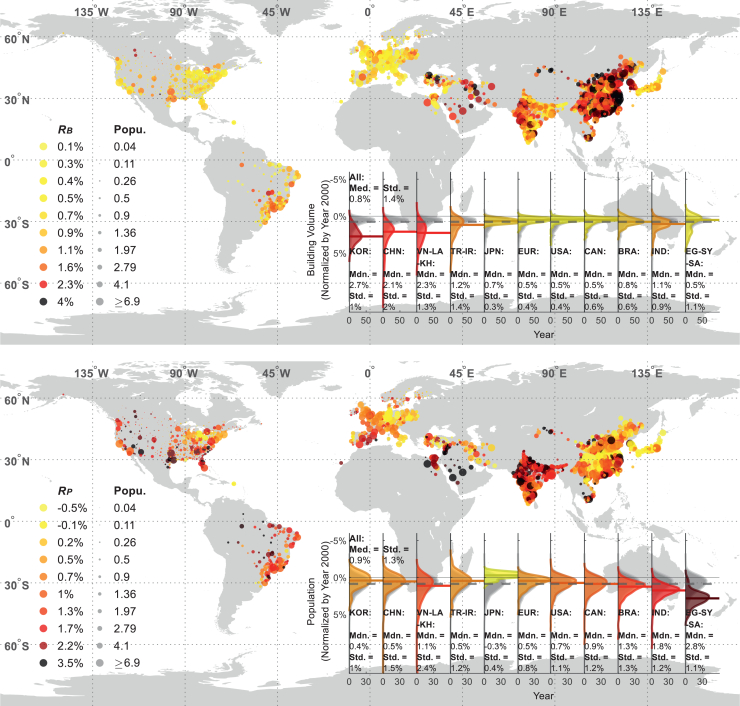
Change rates of building volume (upper) and population (down) sizes across various cities The size of a dot shows the median of the city’s population size during the period.

Figure 1 illustrates the change rates of building volumes and population sizes for various international cities, referring to their values in 2000. These rates are derived as the slopes of linear models (described in the STAR Methods). The reliability of these linear models in capturing building and population trends is given in Figure S1, with *R*^2^ values for population growth exceeding 0.9 in most worldwide cities, and *R*^2^ values for building volume are generally above 0.8 for cities in developing regions (though lower for cities in developed regions, including Europe and the Americas). The building and population trends are further demonstrated in Figures S2 and S3, respectively. These rates are calculated using the Scatterometer Image Reconstruction and the WorldPop database (described in the data and code availability). The building volume and population have increased across worldwide cities, with median change rates exceeding 0 in most regions except Japan. Across all cities under consideration, the median yearly increase rates for building volumes and populations are 0.8% and 0.9%, respectively.

Regions such as South Korea, China, and Southeast Asia (Vietnam-Laos-Cambodia region) exhibit notably high median yearly building volume growth rates exceeding 2.1%, significantly above the global median. Likewise, India, Turkey, and Iran also show relatively high growth, with median rates surpassing 1.1%. Conversely, regions like Europe and North America display modest median growth rates of around 0.5%/year, with Japan and Brazil reaching 0.7% and 0.8% per year, respectively. The variability of building growth rates is reflected by their standard deviations (SD), which tend to be high for the fast-developing countries or regions. For example, South Korea has an SD of 1%, and China’s cities exhibit a higher variability of 2%, mainly due to disparities between its southeastern coastal regions (with rates exceeding 4%) and other areas. In contrast, urbanized regions in Europe, North America, and Japan have lower variability (SD < 0.6%) and growth rates that typically range from 0% to 0.8%.

Population growth rates reveal different trends. Regions such as India, Brazil, Southeast Asia, and parts of the Middle East (Egypt-Syria-Saudi Arabia region) have higher-than-average population growth rates in their cities, with medians surpassing 1.1%. Notably, medians of cities in India and the Egypt-Syria-Saudi Arabia region are even higher than 1.8% and 2.8% per year, respectively. On the other hand, East Asian and European countries show slower population growth, with median rates below 0.5% annually. Cities in Japan and South Korea exhibit stagnating or even declining population trends, indicating outward migration or negative birth rates. Medians of cities in the rest countries are around 0.7%–0.9%/year, aligning with the median of all cities. The SD of most countries or regions ranges from 1% to 1.3%. Notably, population “increase centers”—cities with growth rates exceeding 3.5% annually—tend to be spatially dispersed, except in the southern Middle East. These centers include urban hubs in China, India, the southern United States, and northern Brazil.

### Disproportion levels of building and population changes

**Figure 2:**
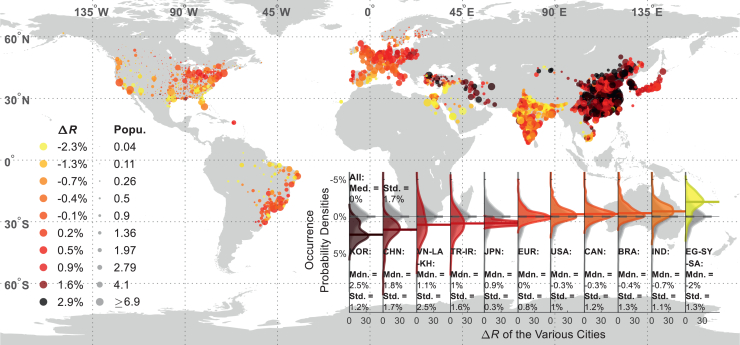
The differences in the building volume and population growth rates (i.e., Δ*R*) for various cities from 2000 to 2020, illustrating the disparity between the two metrics

The inequality between the building volume and population change rates (Δ*R*) indicates whether a city is experiencing over- or underconstruction relative to its population. The Δ*R* value, as shown in Figure 2, tends to be homogeneous across cities within the same country or region, highlighting regional similarities of the city development modes, as well as the positive correlations between building and population growth rates there. These correlations, however, can be different across different countries or regions.

In East Asia, parts of Southeast Asia, and the Middle East, cities tend to have positive Δ*R*, often exceeding the overall median of all cities under consideration. These high Δ*R* values indicate that the building construction rates in cities of these regions surpass the respective population growth rates, and such surpasses are more significant than the cities in Europe and America. Median Δ*R* values in Chinese and South Korean cities are 1.8% and 1.1% per year, respectively, and those in Japan and Southeast Asia exceed 0.9% per year. Interestingly, although building construction rates are moderate (around 1% annually) in many cities across Japan, central Europe, and the northeastern United States, their Δ*R* remains higher than zero because of population stagnation or decline. In contrast, many European cities have Δ*R* values around 0%, suggesting a balance between their building and population growth rates. In the cities of the United States, Canada, and Brazil, building volume growth rates tend to be slightly lower than the respective population growth rates, with median Δ*R* values ranging from −0.4% to −0.3%. In Indian cities, additionally, population growth rates outpace building construction rates, resulting in a median Δ*R* of −0.7% per year. Although Indian cities have relatively high building growth rates (a median of 1.1% annually, higher than many developed countries), their populations grow even faster. A similar pattern is observed in the Egypt-Syria-Saudi Arabia region in the south of the Middle East, where the population increase rates in some cities reach 3.5% per year, leading to a median Δ*R* value of −2% per year.

Variability of Δ*R* of cities in a single country is particularly pronounced in those with extensive spatial area and/or imbalanced building and population growth rates (Δ*R* much higher or lower than 0). For example, cities in China and Southeast Asia exhibit SD values of their Δ*R* as 1.7% and 2.5% per year, respectively, where the city growth rates tend to be faster than the population. Coastal cities in southeastern China, especially, have building growth rates that exceed population growth by over 2.9% per year, deviating significantly from the national median of 1.8%. Likewise, the Δ*R* variability is high in the Egypt-Syria-Saudi Arabia region (SD > 1.3%), where the building construction rates, instead, heavily lag behind their population growth. In contrast, Δ*R* values of cities in Europe and North America exhibit relatively lower variability (SD < 1.2%), with Δ*R* medians close to 0%.

## Discussion

Urbanization over recent decades has been a transformative force, reshaping the environment and societies on a global scale. Previous studies often centered on the problems of urban sprawl and overconstruction, typically measured by increases in developed land area. However, issues such as the vertical urban extension and the need for buildings driven by population growth have received less attention. However, it can be challenging to evaluate whether the per-capita building space is increasing or decreasing and determine if a city is liable to over- or underdevelopment solely by analyzing changes in land area or building volume. This study underscores the importance of integrating population dynamics when assessing the rationality of urban construction speeds. For instance, though Indian cities exhibit moderate building growth rates, these remain insufficient relative to their population growth, resulting in an annual Δ*R* of −0.7%. This disparity highlights the critical need to consider the population changes while evaluating the sustainability of building construction speed.

### Building and population growth rates of country groups

**Figure 3:**
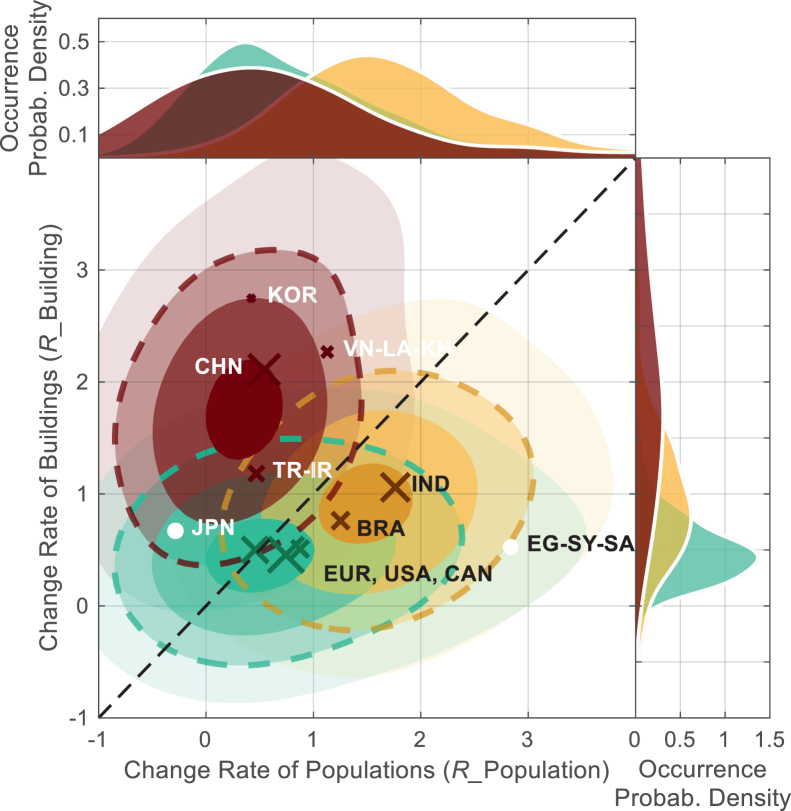
Joint probability densities of building and population growth rates for different countries or regions The levels with different transparencies show the ranges with the increasing rates, with the top 20%, 10%, 5%, and 1% joint probability density for building and population increase rates ranging from −2% to 5%. The contours filled by different colors are determined by cities in various countries or regions (red: China, South Korea, Southeast Asia, Turkey, and Iran; green: Europe, the United States, and Canada; yellow: India and Brazil). Medians of these countries or regions are given as the cross. Cities of the other countries are excluded from the joint probability due to the different joint probability distributions of their rates, and their medians are given as the filled dot.

The results show the similarity in the disproportion levels of the city and population growth within countries, alongside differences across international regions. For each country, though the increasing rates of building volumes may vary across various cities (Figure 1), the differences between the cities’ building and population growth rates (Δ*R*) tend to follow normal distributions, clustering around their corresponding medians (Figure 2). The results agree with the previous findings that the building developments are driven primarily by population growth. This relationship is evidenced by the positive correlations between building and population growth rates (i.e., the *R*_*B*_-*R*_*P*_ relationship), visualized by the joint probability density in Figure 3 and explored in Figures S4 (as grouped distributions) and S5 (as distributions for individual country or region). The greater joint probability densities in Figure 3 correspond to denser dot distributions in Figure S4. Figures 3 and S4 show discrepancies between the change rates of building and population in spite of their positive correlation, and the discrepancies are different in various regions. Specifically, *R*_*B*_-*R*_*P*_ of the Chinese, South Korean, Japanese, and Southeast Asian cities (Vietnam-Laos-Cambodia, VN-LA-KH) distribute above the 45-degree line (*R*_*B*_ = *R*_*P*_), while the dots of the other regions tend to distribute below the 45-degree line.

Ideally, the growth of building volume should align with population growth. The discrepancy between their respective growth rates (Δ*R*) serves as an indicator of proportionality, which is expected to fall within a reasonable range. In practice, however, this “preferred” discrepancy is influenced by a range of factors, including local socioeconomic conditions, urban development strategies, policy frameworks, cultural contexts, and administrative capabilities. This is visible via the different Δ*R* probability densities in different countries or regions in Figure 2. In a Global South country, for example, building volume is expected to grow faster than population as part of efforts to improve living conditions and expand infrastructure. In reality, however, building growth may be constrained or accelerated by local capacities in engineering, governance, and investment.

**Figure 4:**
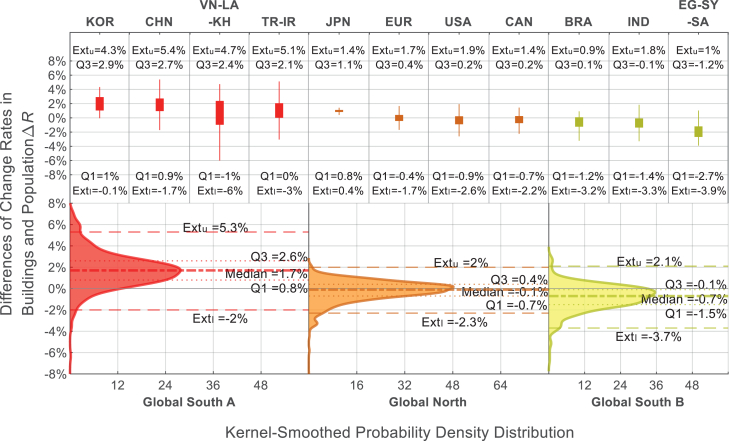
Kernel-smoothed probability density of the difference between building volume and population change rates of the cities (Δ*R*) in various individual or grouped countries (as the upper boxplot) and grouped regions (as the lower surface plot) Global North include Japan, (parts of) Europe, the United States, and Canada, generally showing Δ*R* values close to zero. Global South A includes South Korea, China, Southeast Asia, Turkey, and Iran, characterized by positive Δ*R*. Global South B includes Brazil, India, Egypt, Syria, and Saudi Arabia, characterized by negative Δ*R* values.

Instead of prescribing a universal “optimal” value for Δ*R*, it is more pragmatic to characterize ranges that are typically observed within different development contexts. As illustrated in Figure 4, we grouped the cities into three categories—Global North, Global South A (positive Δ*R*), and Global South B (negative Δ*R*)—and analyzed the kernel-smoothed probability densities of Δ*R* in each group. The distributions reveal distinct tendencies around the group-specific median values. We use the interquartile range (IQR: Q3–Q1) and the upper and lower extremes (i.e., Ext_u_ and Ext_l_, defined as Q1 ± 1.5 × IQR) to suggest ideal and suboptimal ranges of Δ*R*. The identified IQRs are 0.8%–2.6% for Global South A, −1.5% to −0.1% for Global South B, and −0.7% to 0.4% for the Global North. Additionally, Ext_u_ and Ext_l_ are −2.0% to 5.3% for Global South A, −3.7% to 2.1% for Global South B, and −2.3% to 2.0% for the Global North.

These empirical ranges represent the frequently observed values in each group and may reflect what is socially, economically, or politically acceptable under their respective conditions. Notably, both the IQRs and the extremes are considerably wider for Global South cities than for those in the Global North, suggesting greater variability and uncertainty in urban development patterns in developing economies.

### Disproportionate growth driven by socioeconomic factors

**Figure 5:**
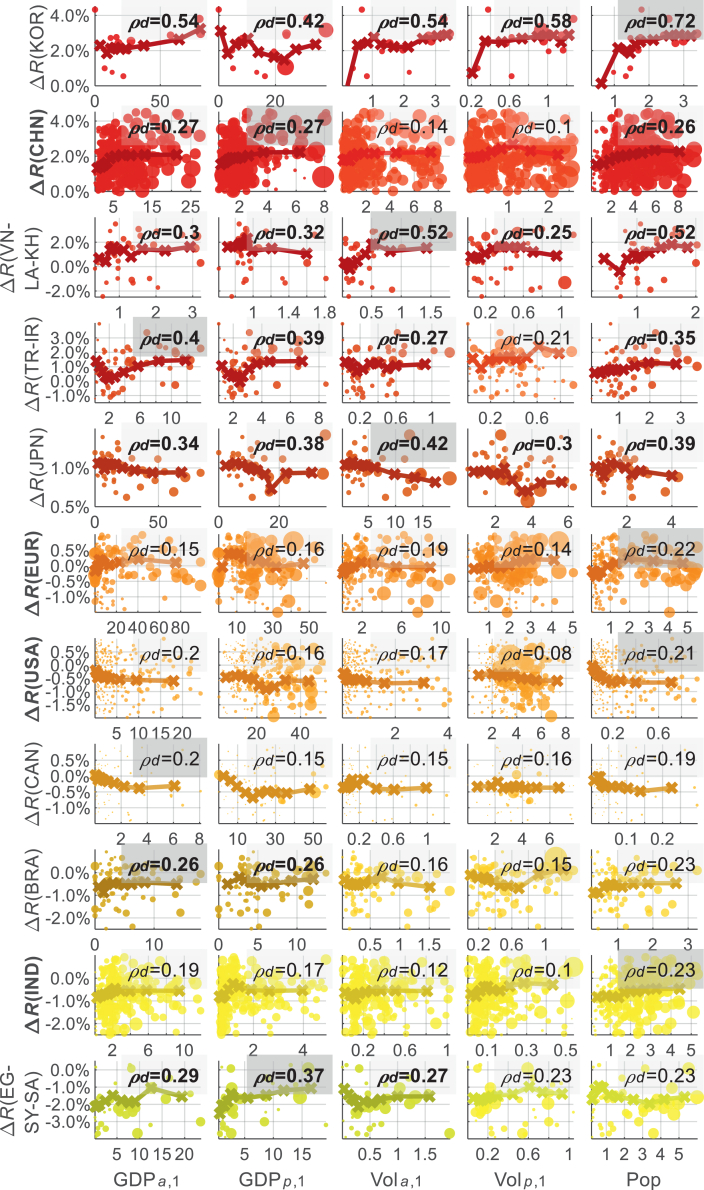
Distributions, smoothed trend lines, and distance correlations (*ρ*_*d*_) of the levels of the disproportion growth and the socioeconomic factors for various countries or regions The independent variables being considered include the total and per-capita purchasing power parity of the gross domestic product (GDP) (GDP_*a*_ and GDP_*p*_, in billion), the total and per-capita building volume (Vol_*a*_ and Vol_*p*_, in power return ratio), and the total population (Pop, in million) within the boundaries of each city in year 2000. Bold font is used in the country or region names with 100 or more cities. Plots are highlighted by a darker color when the distance correlation index is >0.25. For each country, boxes showing the highest *ρ*_*d*_ are highlighted by a dark background color.

The driving forces behind disproportional developments can differ notably across countries or regions. These differences can be visible in Figure 5 via the distributions, trend lines, and distance correlation factors (*ρ*_*d*_) between the differences of the building volume and population increase speeds (i.e., Δ*R*) and several socioeconomic variables. However, no single variable fully explains the variations in Δ*R*, particularly in large countries or country groups, where correlation coefficients remain modest (e.g., *ρ*_*d*_ < 0.21 for the United States, Canada, and Europe; *ρ*_*d*_ < 0.27 for China). This suggests that the mechanisms driving the differences between building and population growth are regionally specific and shaped by complex socioeconomic dynamics. As an important factor related to the city development, the role of gross domestic product (GDP) on Δ*R* of the Global North and South cities (holistically) is investigated in Figure S6.

Among the various socioeconomic and demographic variables examined, economic status (particularly GDP) is a key factor influencing the relative speed of building development with respect to population growth (i.e., Δ*R*). This is evidenced by the relatively high distance correlation coefficients (*ρ*_*d*_) between Δ*R* and both total GDP (GDP_*a*_) and per-capita GDP (GDP_*p*_), as shown in Figure 5, compared to other variables such as building stock or population alone. In Global South regions (e.g., China, Brazil, and Middle Eastern countries), GDP-related variables exhibit stronger correlations with Δ*R*, indicating that cities with higher economic capacity tend to undergo more aggressive building expansion that often outpaces population growth. For instance, in China, the distance correlation between Δ*R* and GDP reaches 0.27. Additionally, Δ*R* is generally more sensitive to GDP_*a*_ than to GDP_*p*_, suggesting that cities with larger economic outputs tend to expand more aggressively (visible for Global South holistically in Figure S6). This trend aligns with speculative or policy-driven development patterns commonly observed in emerging economies.

In contrast, the correlation between Δ*R* and local per-capita building volume remains below 0.14 in China, suggesting that economic development, rather than existing building stock, is a more significant driver of construction intensity in these regions. On the other hand, in Global North regions such as Japan, Europe, and the United States, the relationship is more complex. While population size shows the strongest correlation with Δ*R*, GDP-related factors remain significant. Notably, Δ*R* of the Global North tends to be negatively correlated with GDP_*p*_ (Figure S6), implying that cities with higher per-capita income are more likely to exhibit more balanced or conservative building development than the Global South cities.

Overall, these findings underscore the importance of considering region-specific economic and population status when evaluating the proportionality of urban growth. While GDP serves as a broad indicator of local development potential, its influence on building development aggressiveness differs between emerging and developed economies, offering important implications for urban sustainability and policy design.

### Geospatial connections of disproportionate growth

**Figure 6:**
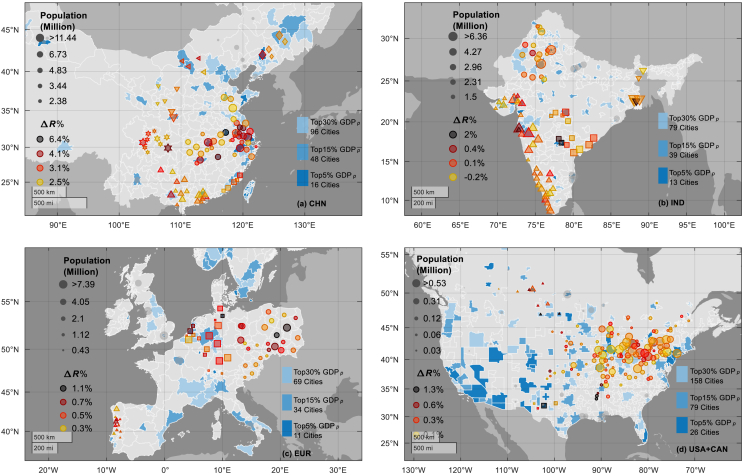
Spatial clustering of cities with the top 30% Δ*R* in China, India, Europe, and North America, where the building growth rates heavily exceed the population growth rate (shown by the colors of dots) The regional economic centers are identified as the cities with the top 5%, 15%, and 30% per-capita GDP (shown by the background colors). Clusters are identified by a density-based clustering algorithm and plotted in different shapes. Details about determining the coefficients of the algorithm are discussed in STAR Methods and further illustrated in Figure S7.

The differences in a city’s building and population growth rates (Δ*R*) can be influenced by the growth patterns of its neighboring cities, driven by competition, cooperation, and economic interdependence. Such interconnections are evident from the spatial clustering of cities where the building growth rates outpace the population growth rate significantly (i.e., cities with top-30% Δ*R*), shown as filled dots in Figure 6. These clusters often form around regional economic centers with the top 5%–10% GDP_*p*_ in each country or region on the figure, though these economic centers do not necessarily exhibit significant growth inequalities. Additionally, some clusters tend to connect with each other due to the closely spaced economic centers or their far-reaching influences.

In developing countries like China and India, clusters of construction-intensive cities often extend toward adjacent clusters (instead of homogeneously in all directions), forming strip-like structures across geographic space. In other words, high-Δ*R* cities can be identified among cities on and in-between the high-speed development belts of the developing countries. For example, in China (Figure 6A), many high-Δ*R* cities cluster around the top-GDP cities that align along the major economic corridor connecting Shanghai, Nanjing, Hefei, Wuhan, and Changsha (with the top-5% GDP_*p*_) from central east to southwest of the country. The rest high-Δ*R* cities in China, though spatially separated, locate around GDP centers and demonstrate directional extension features as well. For instance, clusters in southeastern China along the coast extend northeast toward Shanghai and southwest toward Guangdong. Likewise, clusters around Guangdong extend further west toward the Chengdu-Chongqing city cluster. Similar features are visible in India (Figure 6B), where a high-Δ*R* city cluster forms along the western coastline, guided by top-10% GDP centers such as Ahmedabad, Greater Mumbai, and Bangalore from middle western to southern India. Other clusters, including those around Jaipur in northwestern India and Kolkata-Hyderabad in the center-east of the country, exhibit weaker spatial interconnections.

In contrast to the construction-driven Δ*R* mismatches observed in developing countries, many high-Δ*R* cities in Europe and North America are due to stagnant or declining population growth, rather than accelerated building expansion. This is evident in Figures 6C and 6D, where numerous cities in these regions show positive building volume growth (i.e., *R*_*B*_ > 0) despite low or negative population change rates. In Europe, high-Δ*R* cities are often spatially associated with regions experiencing population decline, as documented in previous studies on the concept of shrinking city.^54^^,^^55^^,^^56^ Similarly, in North America, the high-Δ*R* cities are distributed in the northeastern United States and parts of Canada, corresponding to the shrinking city cluster in the region.^57^ Unlike the contiguous development corridors seen in China or India, high-Δ*R* cities in Europe and North America appear more scattered and lack clear regional aggregation. These spatial patterns tend to be irregular and diffuse, sometimes detached from the presence of nearby economic centers. Importantly, while some high-Δ*R* cities (e.g., in Germany or the northeastern United States) are situated near regions with high GDP, others (in parts of Europe) appear in regions away from the economic centers nearby. This variability highlights the complex interplay between demographic trends, historical legacies, and economic restructuring. In these contexts, the Δ*R* mismatch may not always be the result of overconstruction but rather a by-product of persistent depopulation, deindustrialization, and aging populations—structural issues that differ fundamentally from overconstruction in fast-developing regions.

Taken together, these findings emphasize the need for region-specific interpretations of Δ*R* values. A given Δ*R* magnitude may signal fundamentally different urban dynamics depending on its socioeconomic and demographic context. In Global South regions, high Δ*R* is frequently linked to aggressive urban expansion and speculative development, sometimes in anticipation of future population inflow. By contrast, in the Global North, the same metric often reflects legacy investment inertia or underutilization of existing building stock amid shrinking demand. These distinctions are crucial for understanding the implications of Δ*R*: while high Δ*R* in the Global South may indicate future risks of vacancy or economic inefficiency, in the Global North, it may reflect social or economic transitions that require adaptive reuse and resilience-oriented planning. Addressing the mismatch between building and population growth in shrinking cities, therefore, calls not only for curbing construction but also for integrated strategies addressing housing demand, economic revitalization, and demographic stabilization.

### Limitations of the study

Several limitations should be noted when interpreting the findings of this study. First, although the use of satellite-based datasets for building volume and population enables large-scale, long-term analysis, these datasets may still contain uncertainties due to differences in data sources and sensor capabilities over time. For example, the building volume data were derived from backscatter signals collected by different satellite missions, and despite intersensor calibration, potential inconsistencies in baseline measurements may persist. Similarly, while the WorldPop population estimates are extensively validated, their reliance on diverse data sources and disaggregation models introduces additional uncertainty. Second, city boundaries in this study are based on administrative units, which may not fully reflect functional urban areas or population catchments. Cross-country differences in administrative definitions may also affect intercity comparability. Although administrative levels were selected to ensure consistency within national contexts, using functionality-defined urban extents could yield different spatial patterns. Third, the analysis is conducted at the aggregated city level, which may obscure intracity variations in building construction and population dynamics. Differences between central and peripheral areas, or between formal and informal developments, are not captured in the current framework. Finally, while this study identifies strong associations between building-population growth mismatches and key socioeconomic indicators, it does not explicitly model the broader policy factors that shape urban development. Future research integrating quantitative analysis with case-based approaches may offer deeper insight into the diverse mechanisms underlying mismatched growth patterns across regions.

## Resource availability

### Lead contact

Further information and requests for resources should be directed to and will be fulfilled by the lead contact, Yukai Zou (zou.yukai@outlook.com).

### Materials availability

This study did not generate new unique reagents.

### Data and code availability

• The boundaries of the administrative regions used in this study were obtained from the GADM database (version 4.1, accessed via https://gadm.org/index.html on October 2024) for most countries. Gridded building volume data were obtained from an open-access dataset that is stored in Socioeconomic Data and Applications Center (url: https://www.earthdata.nasa.gov/data/catalog/esdis-ciesin-sedac-uspat-bscatter-1993-2020-1.00 with a doi as https://doi.org/10.7927/GR2E-DH86). Details of the data production method are described in Frolking et al.^58^ Gridded population data were obtained from WorldPop database (https://hub.worldpop.org/geodata/listing?id=64, https://doi.org/10.5258/SOTON/WP00647). Details of the production method are described in Lloyd et al.^59^ Gridded GDP data were acquired from an open-access database (https://zenodo.org/records/7898409, file GDP_2000-2009.7z, https://doi.org/10.5281/zenodo.4350026). Details of the production method are described in Wang and Sun,^60^ with the method given in Eberenz et al.^61^ Rationality of using these datasets is discussed in STAR Methods.
• All original code is available in this article’s supplemental information.
• Any additional information required to reanalyze the data reported in this article is available from the lead contact upon request.

## Acknowledgments

The article is supported by the following grants: 10.13039/501100003453Guangdong Basic and Applied Basic Research Foundation (2024A1515012473), 10.13039/501100001809Natural Science Foundation of China (52308016), Opening Funds of 10.13039/501100015367State Key Laboratory of Building Safety and Built Environment and National Engineering Research Center of Building Technology (BSBE2023-11), and Science and Technology Program of Guangzhou University (PT252022006).

## Author contributions

Conceptualization, D.X. and S.L.; methodology, Y.Z. and S.L.; data collection, S.L.; data analysis plan, Y.Z. and S.L.; data analysis, S.L., Y.H., and Y.Z.; writing – original draft, S.L.; writing – review & editing, S.L. and Y.H.; funding acquisition, S.L., Y.H., and Y.Z.; resources, D.X.; supervision, D.X.

## Declaration of interests

The authors declare no competing interests.

## STAR★Methods

### Key resources table

**Table:** 

REAGENT or RESOURCE	SOURCE	IDENTIFIER
**Deposited data**		

Gridded population counts (Unconstrained global mosaics)	WorldPop	https://hub.worldpop.org/geodata/listing?id=64
Boundaries of the administrative regions	GADM	https://gadm.org/ (version 4.1)
Gridded building volume	Socioeconomic Data and Applications Center (STAR Methods Described in *Scientific Data*)	https://doi.org/10.7927/GR2E-DH86 OR https://www.earthdata.nasa.gov/data/catalog/esdis-ciesin-sedac-uspat-bscatter-1993-2020-1.00
Gridded Gross Domestic Product	Zenodo (STAR Methods Described in *Scientific Data*)	https://zenodo.org/records/7898409 (GDP_2000-2009.7z)

**Software and algorithms**		

MATLAB	MathWorks	https://www.mathworks.com/; RRID: SCR_001622

### Method details

#### Descriptions of the gridded building volume, population, and GDP data

Building volume data were obtained with a spatial resolution of 0.05 degrees in both latitude and longitude.^58^ With a size significantly smaller than most administrative regions (that defines "cities") in this study (Figure S8), the resolution is sufficient for quantifying the city-scale building volumes. This sufficiency is further confirmed by our resolution sensitivity test. In this test, we demonstrate consistency in the city-scale population quantity (Figure S9) and increase rate (Figure S10) that are calculated by the rasterized data in 0.01° and 0.05° resolutions. The building volumes are derived from satellite-based backscatter data collected and processed by the NASA Scatterometer Climate Record Pathfinder (SCP) project. The backscatter data are processed using the Scatterometer Image Reconstruction (SIR) algorithm,^62^ which is reported in the database in rasterized geo-distributions and on a dimensionless decibel scale (dB). A negative value closer to zero means a stronger backscatter, meaning that more of the microwave pulse is scattered back to the satellite sensor, presumably by the built structures with many corner reflectors rather than being scattered away. Here, the data in the decibel scale are converted to the power return ratio for their better linear correlation with independent building volume compared to the decibel scale. According to Frolking et al.,^63^ the database was benchmarked against an independent building volume reference. The benchmark used the gridded building volume data of 215 cities across China, the United States, and Europe. The reference database was built by a random forest model (*R*^2^ > 0.88) that was trained by the data of 25 European, 27 USA, and 24 Chinese cities from government agencies, non-profits, and commercial sources.^64^ Finally, the benchmark indicates that the final gridded building volume product obtained an *R*^2^ of up to 0.70 across 11,548 grids in the urban area. These metrics demonstrate the reliability of the dataset, especially when aggregated at city scales.

Population data were obtained with global coverage and a spatial resolution of 0.01 degrees. The WorldPop database^59^ disaggregates sub-national, census-based population estimates into gridded data using a combination of time-invariant and multi-temporal geospatial predictors. The time-invariant inputs include administrative boundary (from GADM), topography and slope (from Viewfinder Panoramas), roads, pixel area, coastline, waterway (from OpenStreetMap) and average temperature and precipitation during 1970-2000 (from WorldClim). The multi-temporal factors include the night-time light composites (from DMSP-OLS and VIIRS), annual global land cover data (from ESA CCI), the World Database of Protected Areas (from UNEP/IUCN), and built settlement layers combining the JRC Global Human Settlement Layer, the ESA CCI built settlement landcover, and the DLR Global Urban Footprint. These gridded estimates are further calibrated against national and subnational population censuses. We calculated the building volume and population within each city region by summing the values of the corresponding grid points that locates within the defined city boundaries. The accuracy of WorldPop data has been validated in numerous studies. For instance, at the fourth-level (township) administrative scale in China, the *R*^2^ of the gridded population reaches up to 0.906, significantly higher than those of alternative population datasets.^65^ At the second-level administrative scale, the *R*^2^ is close to 1, according to international validation studies.^66^ To further support the reliability of population and building volume metrics used in our analysis, we examined the linear correlation between population and building volume across all city regions, yielding a Pearson correlation coefficient of *ρ* = 0.71. Additionally, we have confirmed that the building volume exhibits a stronger correlation with population than the administrative area does, reinforcing building volume’s relevance as a proxy for urban scale (Figure S11).

Gridded Gross Domestic Product (GDP) data were acquired at a resolution of 30 arc-seconds.^60^ These GDP estimates, expressed in purchasing power parity (PPP), are disaggregated from national (195 countries) and subnational (over 800 provinces or states in 48 countries) statistics using DMSP-OLS nighttime lights and LandScan population distributions, following the method described in.^61^ Validation for the year 2005 shows strong accuracy, with *R*^2^ values of 0.95 and 0.97 for county-level data in China and the United States, respectively—both finer than the administrative units used in our analysis. The reliability of the gridded GDP estimates at the city level is further confirmed by their strong linear relationships to the respective population quantity in most regions across the world (Figure S12).

#### Dividing countries or regions

The task of this study is to quantify the growth rates of building volume and population across 1744 cities over 21 years, from 2000 to 2020. The countries and regions included represent a diverse set of global economics, including Brazil, Canada, China, Europe (mainly West and Central Europe), India, Japan, Middle East, Southeast Asia (Vietnam, Laos, and Cambodia, VN-LA-KH), South Korea, and the United States of America. Countries in the Middle East are divided into two groups, with the first group includes Turkey and Iran (TR-IR), and the other group includes Egypt, Syria, and Saudi Arabia (EG-SY-SA). These countries and regions collectively account for a significant portion of the global population and represent varying stages of urbanization and economic development, thereby enhancing the generalizability of the results. These boundaries represent the areas currently under the control of a country or countries in a region (excluding some regions under disputes), and sovereignty disputes are beyond the scope of this study.

#### Defining city boundaries

Each city's building volume and population should be calculated within its boundaries. Administrative boundaries were used to define city regions to ensure consistency in measuring building volumes and population data. However, administrative systems and region sizes vary across countries. Thus, we adopted different administrative levels for various countries to ensure comparable population sizes of the cities across cities worldwide, as shown in Figure S13. Meanwhile, these cities are large enough to maintain stable population quantities unaffected by daily commuting patterns and small enough to capture inter-regional population flows that are driven by a city’s attractiveness. Table S1 lists the administrative levels we adopted to define a city for each country and their respective city counts. For most countries or regions, the population in a city usually ranges between 1 and 2 million, and is lower than 6 million (given by the median and upper whisker of the boxplot in Figure S13). Though the cities of China and India have been defined by more refined administrative divisions than most countries (e.g., Europe, Japan, South Korea, and those in Southeast Asia), the population sizes of the Chinese and Indian cities are still slightly larger than the other places. In contrast, cities in the USA and Canada generally have smaller population sizes despite incorporating multiple second-level administrative areas. These characteristics of the population sizes are further illustrated by the occurrence probability densities for different regions, which show population peaks at around 2.3 million for Mainland CHN, 1.3 million for cities in the other areas, and below 0.3 million for cities in North America. Only cities with non-zero building volumes, according to the satellite-based measurements, were included in the analysis. The rules we adopted to determine the city boundaries for different countries are specified below.

• Europe, Southeast Asia, Japan, and South Korea: Cities are defined by first-level administrative regions, as further subdivisions often yield excessively small populations or land areas.
• China and India: Cities are defined by the boundaries of second-level administrative regions (prefectures in China and districts in India) to account for their higher population densities.
• North and South America (the United States, Canada, and Brazil): Cities are defined by aggregating second-level administrative regions into Combined Statistical Areas (CSAs) and non-CSA micropolitan statistical areas for the United States,^67^ Census Metropolitan Areas (CMAs) and Census Agglomerations (CAs) for Canada, and Intermediate Geographic Regions (Regiões Geográficas Intermediárias, RGIs) for Brazil.^68^ The aggregation reflects local commuting patterns, economic connections, and the interchanges of goods, employment, and services, as regulated by national statistical agencies. Cities defined as first-level and second-level administration regions can yield vast areas and small populations, respectively.

#### Calculating building volume, population, and GDP of a city

The population, building volume, and Gross Domestic Product (GDP) are determined by aggregating the values of the geo-raster points within the city boundaries. For the population data with a spatial resolution of 0.01 degree, a raster point is regarded as “in the city” if its center falls in the polygon of a city’s boundary. For building volume and GDP with a resolution of 0.05 degrees, however, the percentage of a raster within the boundary is calculated. There can be discontinuities of the original data acquired from multiple sources during the period as long as 21 years, though they are organized in the same dataset. The data building volumes, for example, are acquired by three satellites that operate in 1993-2000, 1999-2009, and 2007-2020. The building volume before and after changing the satellite are aligned according to the differences of the data from the two sources in the overlapping periods, by shifting the data before or after the period up or down a constant in case of their different zero offsets. Likewise, the population is fixed according to the data in the census years (2000 and 2010), and the values of the rest years are shifted by a constant to align those in the census years.

#### Calculating growth rates and disproportion metrics

Whether a city's building volume and population growths are proportionate is quantified by the differences between its building and population change rates (Equation 1) since the buildings are mainly constructed to meet the spatial needs of humans. Significant positive or negative values of Δ*R* indicate over-construction or under-construction relative to the respective population growth. The change rates are defined as the slopes (*R*_*B*_ and *R*_*P*_) of the normalized building volume (*B*) and population (*P*) over the 21 years, as described in Equations 2 and 3, respectively. These slopes are calculated using robust regression, where the year (*y*) is the independent variable and *B* or *P* is the dependent variable. Coefficients *B*_0_ and *P*_0_ are the intercepts also determined by the regression. Normalizations of the building volume and population are performed by dividing their values in each city by their respective baselines in the year 2000 to allow for international comparisons across regions with differing development baselines.

(Equation 1) $$\Delta R=R_{B}-R_{P}$$

(Equation 2) $$B=R_{B}y+B_{0}$$

(Equation 3) $$P=R_{p}y+P_{0}$$

#### Calculating distance correlation

The distance correlation *ρ*_*d*_ is adopted to assess the relationship between growth disparities (Δ*R*) and socioeconomic factors such as GDP, population size, and per-capita building volume across different regions. The correlation *ρ*_*d*_ is zero if the vectors are independent. Distance correlation measures both linear and nonlinear associations between the paired variables. Unlike Pearson correlation, distance correlation captures both linear and non-linear associations between paired variables.

The distance correlation of two variables, *X* and *Y*, is mathematically defined as:

(Equation 4) $$\rho_{d}=\frac{\text{dCov}\left(X,Y\right)}{\sqrt{\text{dVar}\left(X\right)\text{dVar}\left(Y\right)}}$$

Here, dCov(*X*, *Y*) represents the distance covariance of variables *X* and *Y*. dVar(*X*) and dVar(*Y*) are the distance variances of variables *X* and *Y*, respectively. dVar represents a case when the two variables of dCov are identical. The squared dCov(*X*, *Y*) are calculated as follows:

(Equation 5) $${\text{dCov}}^{2}\left(X,Y\right)=\frac{1}{n^{2}}\sum_{j=1}^{n}\sum_{k=1}^{n}A_{j,k}B_{j,k}$$

(Equation 6) $$A_{j,k}=a_{j,k}-{\bar{a}}_{j\cdot }-{\bar{a}}_{\cdot k}+{\bar{a}}_{\cdot \cdot }$$

(Equation 7) $$B_{j,k}=b_{j,k}-{\bar{b}}_{j\cdot }-{\bar{b}}_{\cdot k}+{\bar{b}}_{\cdot \cdot }$$

Here, *a*_*j,k*_ and *b*_*j,k*_ represent values of two *n*-by-*n* matrixes in the *j*th row and *k*th column that are determined by, respectively, *X* and *Y*. Additionally, *ā*_*j*·_ represents the average of the *j*th row, *ā*_·*k*_ represents the average of the *k*th column, and *ā*·· represents the average of the entire distance matrix of the matrix. Specifically, *a*_*j,k*_ and *b*_*j,k*_ can be calculated as follows, where ||·|| denotes the Euclidean norm (distance).

(Equation 8) $$a_{j,k}=\left\|X_{j}-X_{k}\right\|$$

(Equation 9) $$b_{j,k}=\left\|Y_{j}-Y_{k}\right\|$$

#### Calculating the trend line of Δ*R* and socioeconomic factors by smoothing

For each subplot in Figure 5, the values constructing the smoothed trend line are calculated at various percentiles of the socioeconomic variable under consideration (Var), ranging from 2.5th to 97.5th in 5% intervals. At each interval of the variable under consideration (Var_*i*%_), the inequalities (Δ*R*) are calculated by three factors.

The first is the proximity of the socioeconomic variable under investigation (Var) to a series of its values (Var_*i*%_). In other words, Δ*R* in various Var_*i*%_ values are averaged by Δ*R* of cities with approximate values of Var to Var_*i*%_. The proximity is scaled by the weights derived from the normal distribution *f*(Var, *μ*, *σ*). Here, *μ* stands for the median of the distribution *f*(Var, *μ*, *σ*), and *σ*^2^ is the variance. For each Var_*i*%_, its *μ* takes the value of Var_*i*%_ so that the weight (determined partially by *f*(Var, *μ*, *σ*)) peaks at each Var_*i*_ under consideration. The standard deviation *σ* is calculated as (1/0.05) times the standard deviation (StD) according to the Δ*R* of the cities with their Var ranging from Var_*i*%_ – 2.5% to Var_*i*%_ + 2.5%. In this case, the normal distribution can be effective mainly in the range of Var_*i*%_ ± 2.5%, and reduces fast when the Var gets away from Var_*i*%_, with a speed that adapts to the StD of the Var values around the Var_*i*%_ under investigation.

Apart from proximity, the weights are further scaled by the cities’ population and the coefficient of determination of the linear models in depicting the building and population changes during the 21 years. These weight settings prioritize well-populated regions where the building volume and population changes are well-depicted by their respective change rates. The weights are finally rescaled in the range of 0 to 1.

#### Clustering analysis

Cities with the top 30% ΔR in a region are clustered by the DBSCAN (Density-Based Spatial Clustering of Applications with Noise) algorithm according to their distances. The DBSCAN algorithm relies on two user-defined parameters that are set according to the recommendations of DBSCAN developers and previous users.

The minimum number of neighbors required for a core point (**MinPts**): Set to four, ensuring a cluster consists of at least four cities.

The neighborhood search radius (**Epsilon**): Epsilon affects the inter-cluster distance and the number of identified clusters. If Epsilon is too small, larger portions of the dataset will be classified as outliers and remain unclustered; if it is too large, clusters may merge together. Epsilon is determined as the ‘knee point' of the *k*-distance plot (Figure S7), which is each city's distance to its *k*th nearest neighbor, sorted in ascending order. The knee point represents the place where the *k*-distance starts to increase significantly. Epsilon is determined by the *k*-distance value around the knee point that separates the outlying cities from non-outliers with high distances from the neighbors. In this study, *k* is set to four, equal to MinPts, following the common practice in previous studies. The knee point is selected by the following steps.

1. Walk along the *k*-distance plot one bisection point at a time.
2. Fitting two lines, one to all the points to the left of the bisection point and one to all the points to the right.
3. Exclude distances higher than the [third quantile + 3 × inter-quantile range] (in case the selection being dominated by one or two extreme distances). Theoretically, the knee should be selected as the bisection point that minimizes the sum of the absolute errors for the two fits (right-side and left-side). However, for each region described in Figure 6, a few adjustments were made on the knee point for rational clusters, and the influences are as follows:
  • **CHN:** The adopted knee point is 5 ranks higher than the one identified by the minimum absolute error algorithm, resulting in a shift from 2.106 to 2.450. This change allows the ‘▼' cluster in Figure 6A to be recognized instead of being considered an outlier, without affecting other clusters.
  • **IND:** The point identified by the minimum absolute error algorithm is adopted, resulting in Epsilon = 2.561. A lower Epsilon would separate the single cluster marked as ‘▪' in Figure 6B into two distinct clusters, indicating that cities in this cluster are developed around two centers and merged. Moreover, the northeast cluster marked as ‘▼' will be recognized as an outlier.
  • **EUR:** The adopted point is 5 ranks lower than the one identified by the minimum-error method, shifting Epsilon from 2.521 to 2.109. This change helps distinguish two clusters in central Europe (marked as ‘•' around Berlin and '▪' around Amsterdam) in Figure 6C, preventing them from merging into a single cluster. The cluster(s), merged or separated, are in round and irregular shapes without directional extensions like those in developing countries.
  • **USA and CAN:** The adopted point is 5 ranks lower than the one identified by the minimum-absolute-error method, shifting Epsilon from 2.976 to 2.679. This adjustment allows a few cities to the northwest of the largest clusters in Figure 6D to be recognized as an individual cluster marked as ‘▲,' without affecting the geospatial shape of the largest cluster in the northeast United States.

### Quantification and statistical analysis

All data processing analyses and figure plotting were conducted using MATLAB.

## Supplemental information

Document S1. Figures S1–S13 and Table S1

Data S1. Code developed to extract data from the data sources, process the data, and plot the figures

## References

[1] Xu G., Zhou Z., Jiao L., Zhao R. (2020). Compact Urban Form and Expansion Pattern Slow Down the Decline in Urban Densities: A Global Perspective. Land Use Policy.

[2] Xu G., Dong T., Cobbinah P.B., Jiao L., Sumari N.S., Chai B., Liu Y. (2019). Urban expansion and form changes across African cities with a global outlook: Spatiotemporal analysis of urban land densities. J. Clean. Prod..

[3] Masini E., Tomao A., Barbati A., Corona P., Serra P., Salvati L. (2019). Urban Growth, Land-use Efficiency and Local Socioeconomic Context: A Comparative Analysis of 417 Metropolitan Regions in Europe. Environ. Manag..

[4] Koroso N.H., Lengoiboni M., Zevenbergen J.A. (2021). Urbanization and urban land use efficiency: Evidence from regional and Addis Ababa satellite cities, Ethiopia. Habitat Int..

[5] Xu L., Huang Q., Ding D., Mei M., Qin H. (2018). Modelling urban expansion guided by land ecological suitability: A case study of Changzhou City, China. Habitat Int..

[6] Lin T., Xue X., Shi L., Gao L. (2013). Urban spatial expansion and its impacts on island ecosystem services and landscape pattern: A case study of the island city of Xiamen, Southeast China. Ocean Coast Manag..

[7] Chettry V. (2023). A Critical Review of Urban Sprawl Studies. J. Geovis. Spat. Anal..

[8] Kumar A., Pandey A.C., Hoda N., Jeyaseelan A.T. (2011). Evaluation of urban sprawl pattern in the tribal-dominated cities of Jharkhand state, India. Int. J. Rem. Sens..

[9] Wang Q., Wang H. (2022). Spatiotemporal dynamics and evolution relationships between land-use/land cover change and landscape pattern in response to rapid urban sprawl process: A case study in Wuhan, China. Ecol. Eng..

[10] Fahad S., Li W., Lashari A.H., Islam A., Khattak L.H., Rasool U. (2021). Evaluation of land use and land cover Spatio-temporal change during rapid Urban sprawl from Lahore, Pakistan. Urban Clim..

[11] Lou S., Feng C., Zhang D., Zou Y., Huang Y. (2024). Heat exposure inequalities in Hong Kong from 1981 to 2021. Urban Clim..

[12] Zhang Y., Wang L., Tang Z., Zhang K., Wang T. (2022). Spatial effects of urban expansion on air pollution and eco-efficiency: Evidence from multisource remote sensing and statistical data in China. J. Clean. Prod..

[13] Hien P.D., Men N.T., Tan P.M., Hangartner M. (2020). Impact of urban expansion on the air pollution landscape: A case study of Hanoi, Vietnam. Sci. Total Environ..

[14] Wang M., Fu X., Zhang D., Lou S., Li J., Chen F., Li S., Tan S.K. (2023). Urban agglomeration waterlogging hazard exposure assessment based on an integrated Naive Bayes classifier and complex network analysis. Nat. Hazards.

[15] Navamuel E.L., Rubiera Morollón F., Moreno Cuartas B. (2018). Energy consumption and urban sprawl: Evidence for the Spanish case. J. Clean. Prod..

[16] Frenkel A., Israel E. (2018). Spatial inequality in the context of city-suburb cleavages–Enlarging the framework of well-being and social inequality. Landsc. Urban Plann..

[17] Seto K.C., Fragkias M., Güneralp B., Reilly M.K. (2011). A Meta-Analysis of Global Urban Land Expansion. PLoS One.

[18] Luo J., Zhang X., Wu Y., Shen J., Shen L., Xing X. (2018). Urban land expansion and the floating population in China: For production or for living?. Cities.

[19] Schneider A., Mertes C.M. (2014). Expansion and growth in Chinese cities, 1978–2010. Environ. Res. Lett..

[20] Huang Q., Liu Y. (2021). The Coupling between Urban Expansion and Population Growth: An Analysis of Urban Agglomerations in China (2005–2020). Sustainability.

[21] Li G., Sun S., Fang C. (2018). The varying driving forces of urban expansion in China: Insights from a spatial-temporal analysis. Landsc. Urban Plann..

[22] Mahtta R., Fragkias M., Güneralp B., Mahendra A., Reba M., Wentz E.A., Seto K.C. (2022). Urban land expansion: the role of population and economic growth for 300+ cities. npj Urban Sustain..

[23] Jia M., Liu Y., Lieske S.N., Chen T. (2020). Public policy change and its impact on urban expansion: An evaluation of 265 cities in China. Land Use Policy.

[24] Mahtta R., Mahendra A., Seto K.C. (2019). Building up or spreading out? Typologies of urban growth across 478 cities of 1 million+. Environ. Res. Lett..

[25] Jiang Y., Waley P. (2023). Keeping up with the zones(es): how competing local governments in China use development zones as back doors to urbanization. Urban Geogr..

[26] Li Q., Xu Y., Yang X., Chen K. (2023). Unveiling the Regional Differences and Convergence of Urban Sprawl in China, 2006–2019. Land.

[27] Shi L., Leichtle T., Wurm M., Taubenböck H. (2022). The “ghost neighborhood” phenomenon in China—geographic locations and intra-urban spatial patterns. Environ. Plan. B Urban Anal. City Sci..

[28] Gu R., Xie Z., Takatori C., Herold H., Xie X. (2023). To What Extent Can Satellite Cities and New Towns Serve as a Steering Instrument for Polycentric Urban Expansion during Massive Population Growth?—A Comparative Analysis of Tokyo and Shanghai. ISPRS Int. J. GeoInf..

[29] Yin G., Liu Y., Chen Y. (2024). “Ghost city” or habitable city? The production and transformation of space in China's new towns. Cities.

[30] Audirac I. (2018). Introduction: Shrinking Cities from marginal to mainstream: Views from North America and Europe. Cities.

[31] Hartt M.D. (2018). How cities shrink: Complex pathways to population decline. Cities.

[32] Wang L., Fan H., Wang Y. (2019). An estimation of housing vacancy rate using NPP-VIIRS night-time light data and OpenStreetMap data. Int. J. Rem. Sens..

[33] Gao J., Zhong X., Cai W., Ren H., Huo T., Wang X., Mi Z. (2019). Dilution effect of the building area on energy intensity in urban residential buildings. Nat. Commun..

[34] Luyi T. (2020). A review on definitions and measurements for urban expansion. World Reg. Stud..

[35] Huang C., Xu N. (2022). Quantifying urban expansion from 1985 to 2018 in large cities worldwide. Geocarto Int..

[36] Jiao L., Mao L., Liu Y. (2015). Multi-order Landscape Expansion Index: Characterizing urban expansion dynamics. Landsc. Urban Plann..

[37] Jiao L., Liu J., Xu G., Dong T., Gu Y., Zhang B., Liu Y., Liu X. (2018). Proximity Expansion Index: An improved approach to characterize evolution process of urban expansion. Comput. Environ. Urban Syst..

[38] Fenglei F., Wei F. (2014). Understanding spatial-temporal urban expansion pattern (1990–2009) using impervious surface data and landscape indexes: a case study in Guangzhou (China). J. Appl. Remote Sens..

[39] Zambon I., Colantoni A., Salvati L. (2019). Horizontal vs vertical growth: Understanding latent patterns of urban expansion in large metropolitan regions. Sci. Total Environ..

[40] Zhou Y., Li X., Chen W., Meng L., Wu Q., Gong P., Seto K.C. (2022). Satellite mapping of urban built-up heights reveals extreme infrastructure gaps and inequalities in the Global South. Proc. Natl. Acad. Sci. USA.

[41] Abd Elraouf R., Elmokadem A., Megahed N., Abo Eleinen O., Eltarabily S. (2022). The impact of urban geometry on outdoor thermal comfort in a hot-humid climate. Build. Environ..

[42] Chen G., Rong L., Zhang G. (2021). Impacts of urban geometry on outdoor ventilation within idealized building arrays under unsteady diurnal cycles in summer. Build. Environ..

[43] Zhou S., Shi T., Li S., Dong Y., Sun J. (2023). The impact of urban morphology on multiple ecological effects: Coupling relationships and collaborative optimization strategies. Build. Simulat..

[44] Xia C., Yeh A.G.-O., Zhang A. (2020). Analyzing spatial relationships between urban land use intensity and urban vitality at street block level: A case study of five Chinese megacities. Landsc. Urban Plann..

[45] Correia Filho W.L.F., Oliveira-Júnior J.F.d., Santos C.T.B.d., Batista B.A., Santiago D.d.B., Silva Junior C.A.d., Teodoro P.E., Costa C.E.S.d., Silva E.B.d., Freire F.M. (2022). The influence of urban expansion in the socio-economic, demographic, and environmental indicators in the City of Arapiraca-Alagoas, Brazil. Remote Sens. Appl.: Soc. Environ..

[46] Ren X., Ye L. (2018). Urbanization and Urban Governance in China: Issues, Challenges, and Development.

[47] Wu F., Zhang F., Webster C. (2013). Informality and the Development and Demolition of Urban Villages in the Chinese Peri-urban Area. Urban Stud..

[48] Zhao L., Liu X., Xu X., Liu C., Chen K. (2022). Three-Dimensional Simulation Model for Synergistically Simulating Urban Horizontal Expansion and Vertical Growth. Remote Sens..

[49] Handayani H.H., Murayama Y., Ranagalage M., Liu F., Dissanayake D. (2018). Geospatial Analysis of Horizontal and Vertical Urban Expansion Using Multi-Spatial Resolution Data: A Case Study of Surabaya, Indonesia. Remote Sens..

[50] Yang C., Zhao S. (2022). Urban vertical profiles of three most urbanized Chinese cities and the spatial coupling with horizontal urban expansion. Land Use Policy.

[51] Wang N., Chen Z., Li T., Zhen M. (2022). Spatiotemporal Pattern Evolution and Influence Mechanism of Urban Vertical Expansion: A Case Study of Jiangsu Province, China. Land.

[52] Liu X., Wu X., Li X., Xu X., Liao W., Jiao L., Zeng Z., Chen G., Li X. (2025). Global Mapping of Three-Dimensional (3D) Urban Structures Reveals Escalating Utilization in the Vertical Dimension and Pronounced Building Space Inequality. Engineering.

[53] Ustaoglu E., Williams B. (2017). Determinants of Urban Expansion and Agricultural Land Conversion in 25 EU Countries. Environ. Manag..

[54] Wolff M. (2018). Understanding the role of centralization processes for cities – Evidence from a spatial perspective of urban Europe 1990–2010. Cities.

[55] Newsham N., Rowe F. (2023). Understanding trajectories of population decline across rural and urban Europe: A sequence analysis. Popul. Space Place.

[56] Cortinovis C., Geneletti D., Haase D. (2022). Higher immigration and lower land take rates are driving a new densification wave in European cities. npj Urban Sustain..

[57] Murdoch III J. (2018). Specialized vs. diversified: The role of neighborhood economies in shrinking cities. Cities.

[58] Frolking S., Milliman T., Mahtta R., Paget A., Long D.G., Seto K.C. (2022).

[59] Lloyd C.T., Chamberlain H., Kerr D., Yetman G., Pistolesi L., Stevens F.R., Gaughan A.E., Nieves J.J., Hornby G., MacManus K. (2019). Global spatio-temporally harmonised datasets for producing high-resolution gridded population distribution datasets. Big Earth Data.

[60] Wang T., Sun F. (2022). Global gridded GDP data set consistent with the shared socioeconomic pathways. Sci. Data.

[61] Eberenz S., Stocker D., Röösli T., Bresch D.N. (2020). Asset exposure data for global physical risk assessment. Earth Syst. Sci. Data.

[62] Long D.G. (2017). Comparison of SeaWinds Backscatter Imaging Algorithms. IEEE J. Sel. Top. Appl. Earth Obs. Rem. Sens..

[63] Frolking S., Milliman T., Mahtta R., Paget A., Long D.G., Seto K.C. (2022). A global urban microwave backscatter time series data set for 1993–2020 using ERS, QuikSCAT, and ASCAT data. Sci. Data.

[64] Li M., Koks E., Taubenböck H., van Vliet J. (2020). Continental-scale mapping and analysis of 3D building structure. Remote Sens. Environ..

[65] Bai Z., Wang J., Wang M., Gao M., Sun J. (2018). Accuracy Assessment of Multi-Source Gridded Population Distribution Datasets in China. Sustainability.

[66] Liu L., Cao X., Li S., Jie N. (2024). A 31-year (1990–2020) global gridded population dataset generated by cluster analysis and statistical learning. Sci. Data.

[67] Bureau U.S.C., USCB (2023). Core based statistical areas (CBSAs), metropolitan divisions, and combined statistical areas (CSAs).

[68] IBGE. (2021). Municipal Digital Mesh of the Brazilian Political-Administrative Division.

