# Supplementary material for: Mapping the mismatch between building and population growth: A global study of 1,700 cities
Source: iScience. 2025 Aug 5;28(9):113289. doi: 10.1016/j.isci.2025.113289 (PMC12396097; doi:10.1016/j.isci.2025.113289)
Supplement: Document S1. Figures S1–S13 and Table S1 [file mmc1.pdf]

iScience, Volume *28*

Supplemental information

Mapping the mismatch between building and population growth: A global study of 1,700 cities

Siwei Lou (娄驷渭), Yu Huang (黄宇), Yukai Zou (邹煜凯), and Dawei Xia (夏大为)

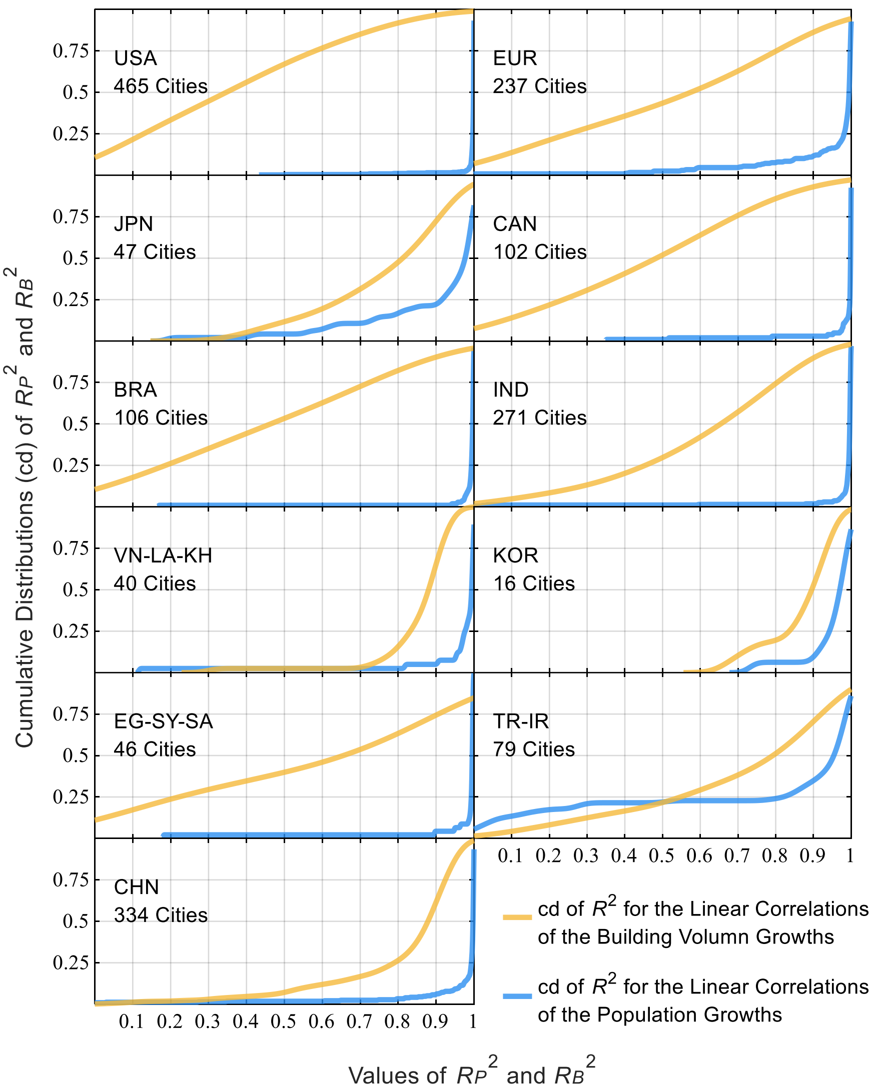

Figure S1. Cumulative probability distributions of R^2 (smoothed by Kernel function) for the linear models of normalized building volume and population growth in global cities (2000-2020).

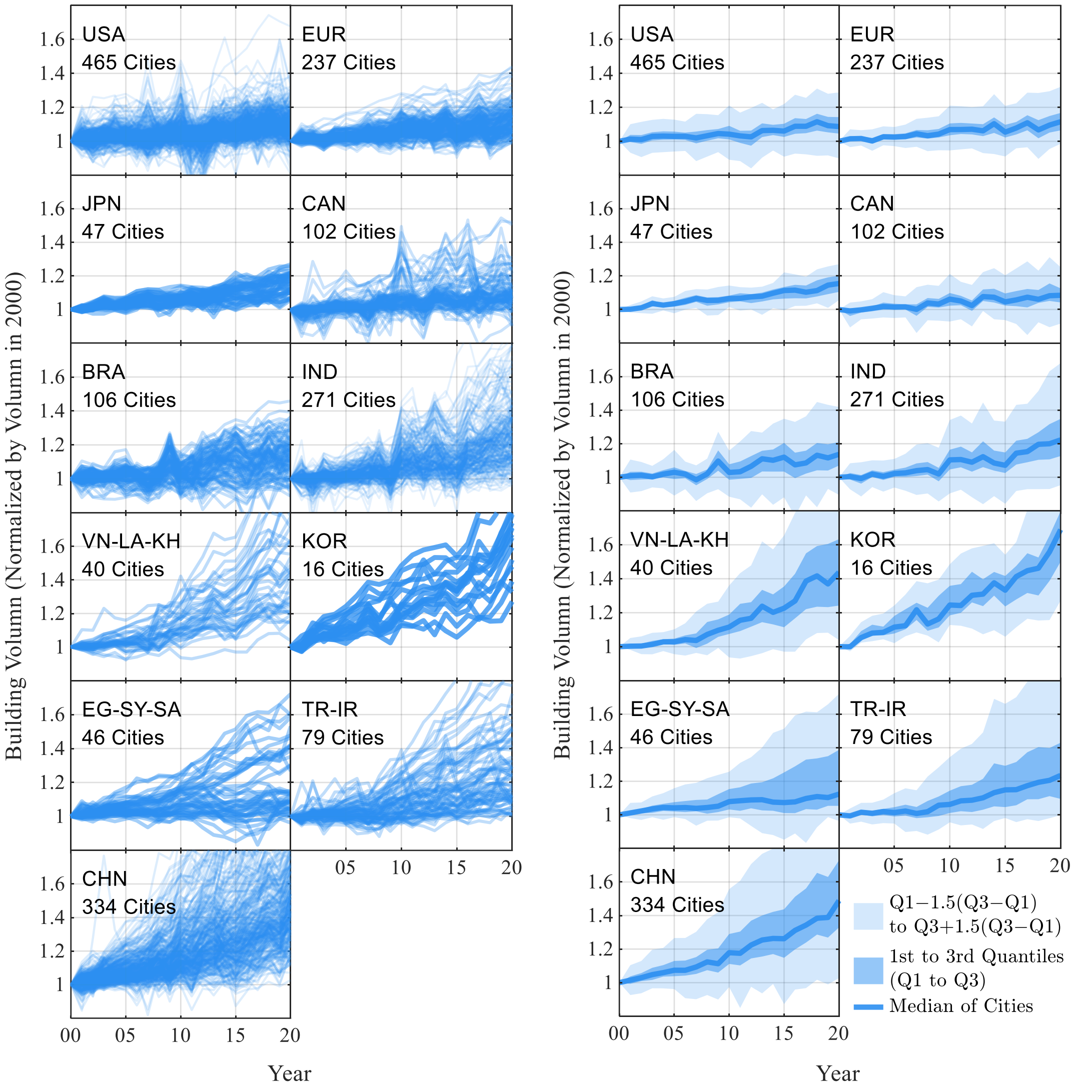

Figure S2. Distributions (left) and the median, interquartile range, and non-outlier extremes (right) of normalized building volume across global cities from 2000 to 2020.

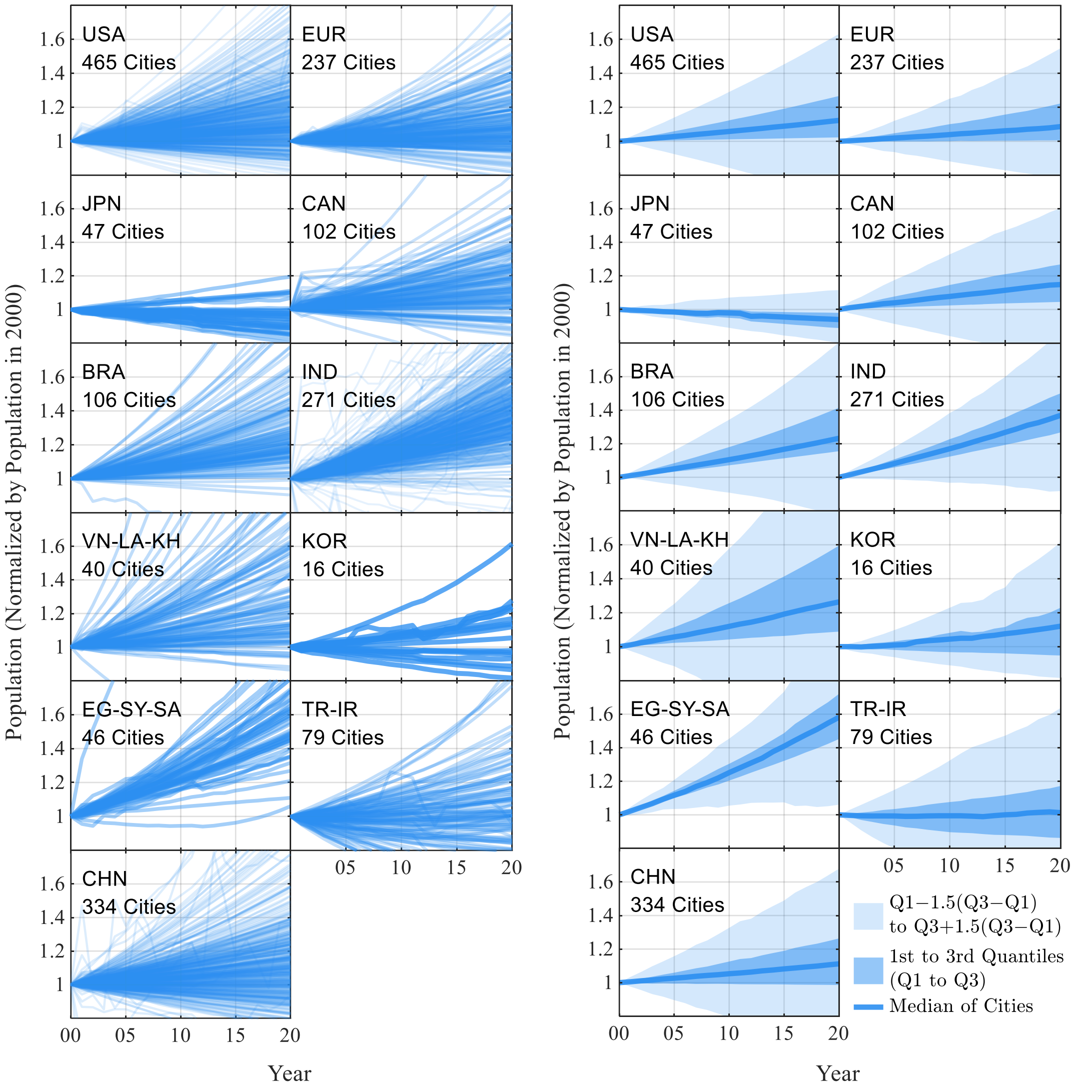

Figure S3. Distributions (left) and the median, interquartile range, and non-outlier extremes (right) of normalized population sizes across global cities from 2000 to 2020.

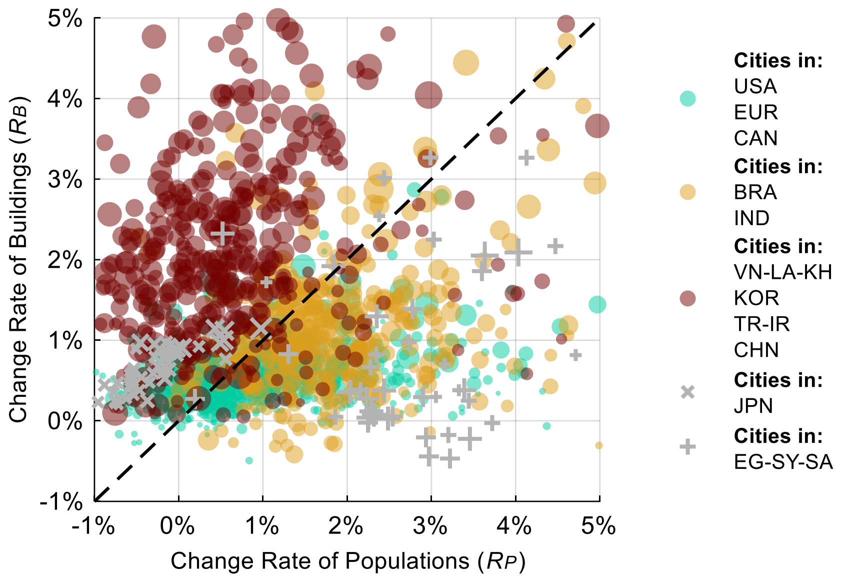

Figure S4. Scatterplot of normalized building and population growth rates across city groups defined in Figure 3, with ungrouped cities from JPN and EG–SY–SA shown separately.

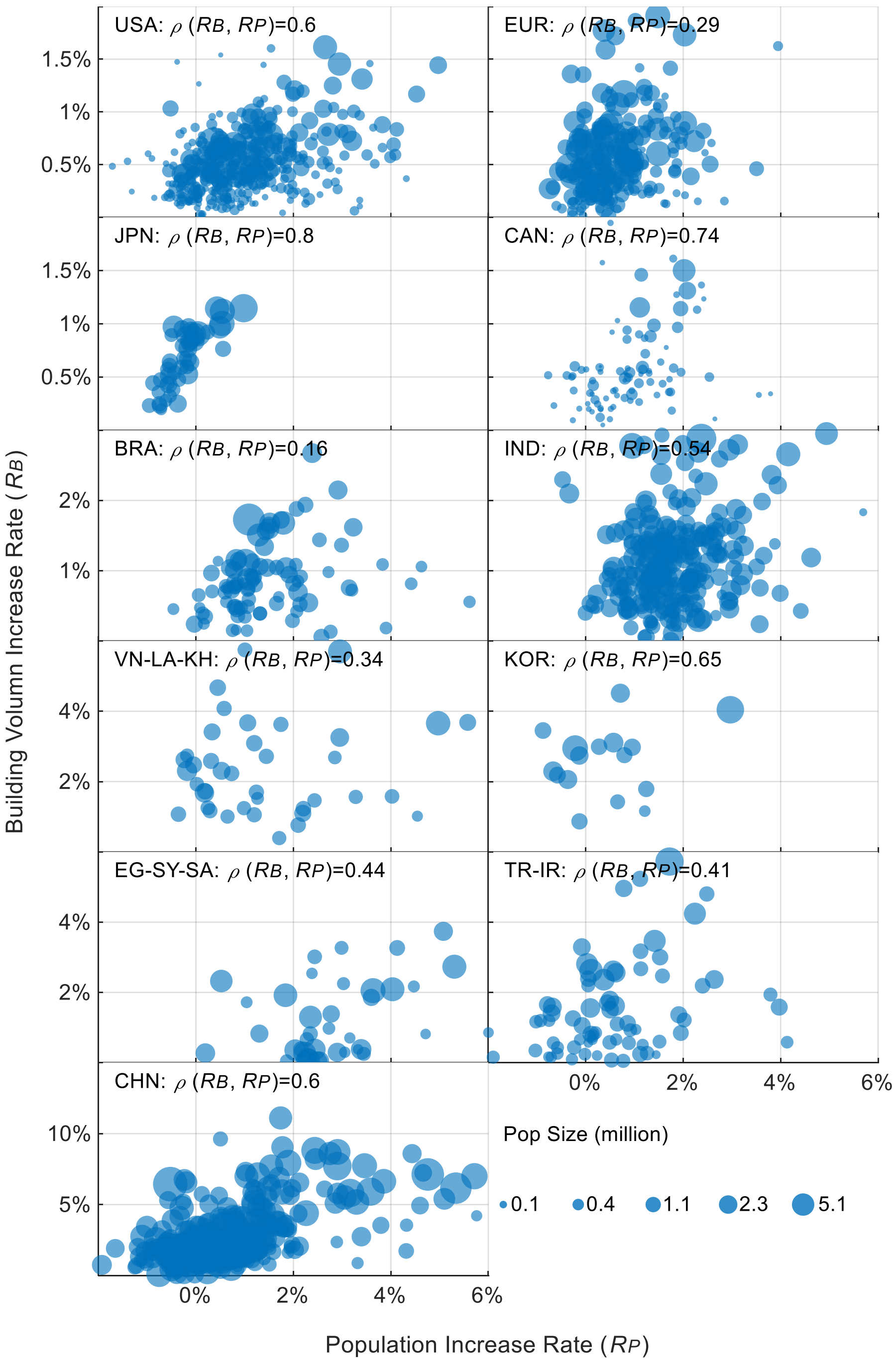

Figure S5. Distributions of normalized building and population growth rates across cities in different countries or regions, with weighted Pearson correlation coefficients (ρ) based on R^2 values of the linear trend model and the city population size.

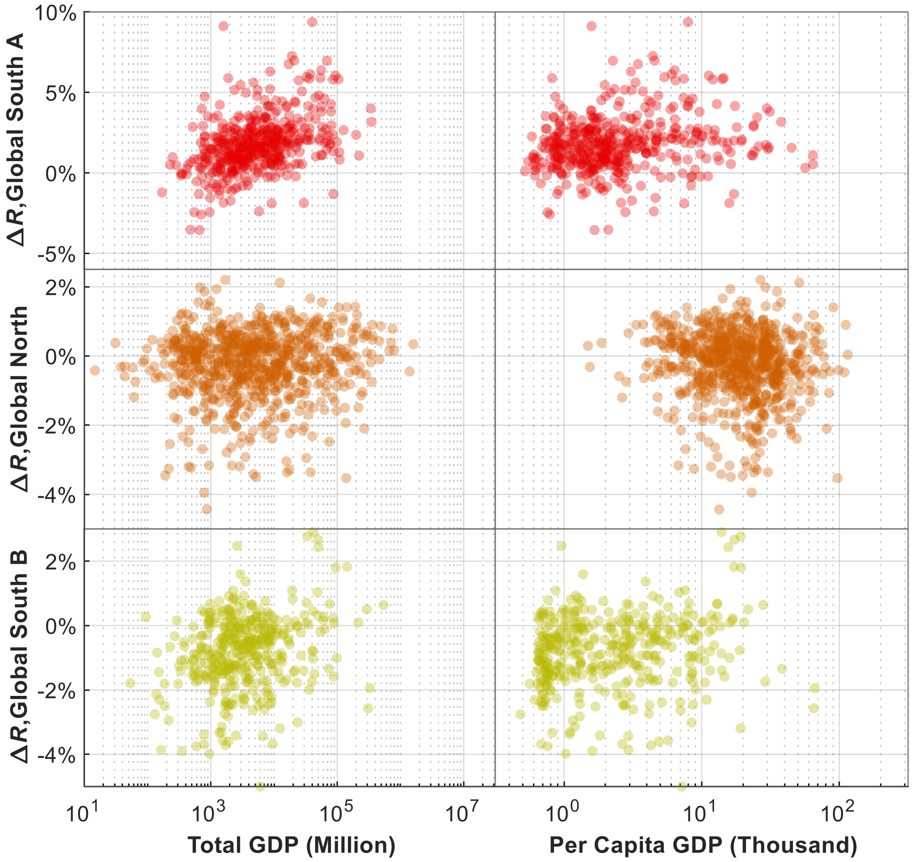

Figure S6. Associations of total and per-capita GDP with the difference between building and population growth rates (ΔR) across cities in Global North and Global South groups A and B.

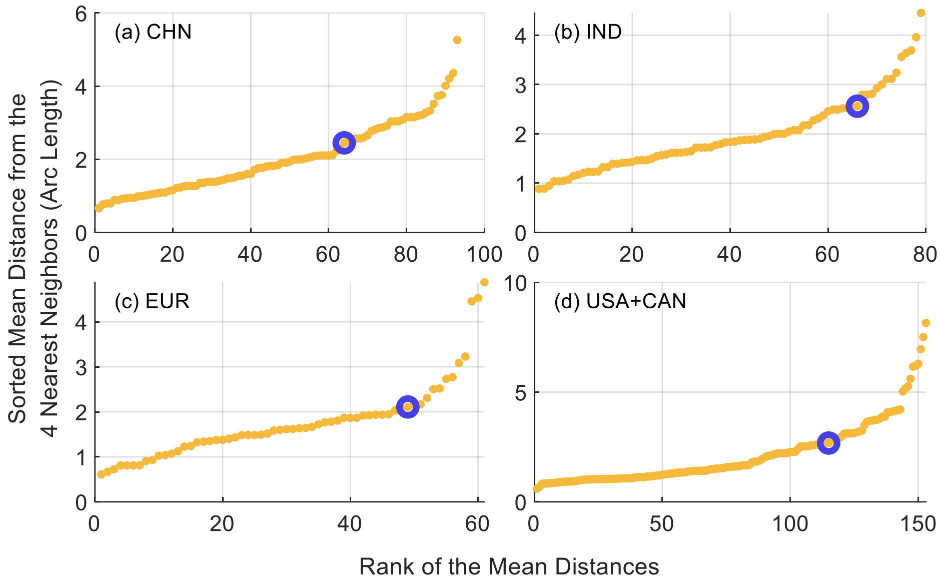

Figure S7. K-distance plots (4th nearest neighbor) for cities in Figure 6, with knee points used to determine Epsilon values shown as blue circles and excessively large distances (outliers) excluded.

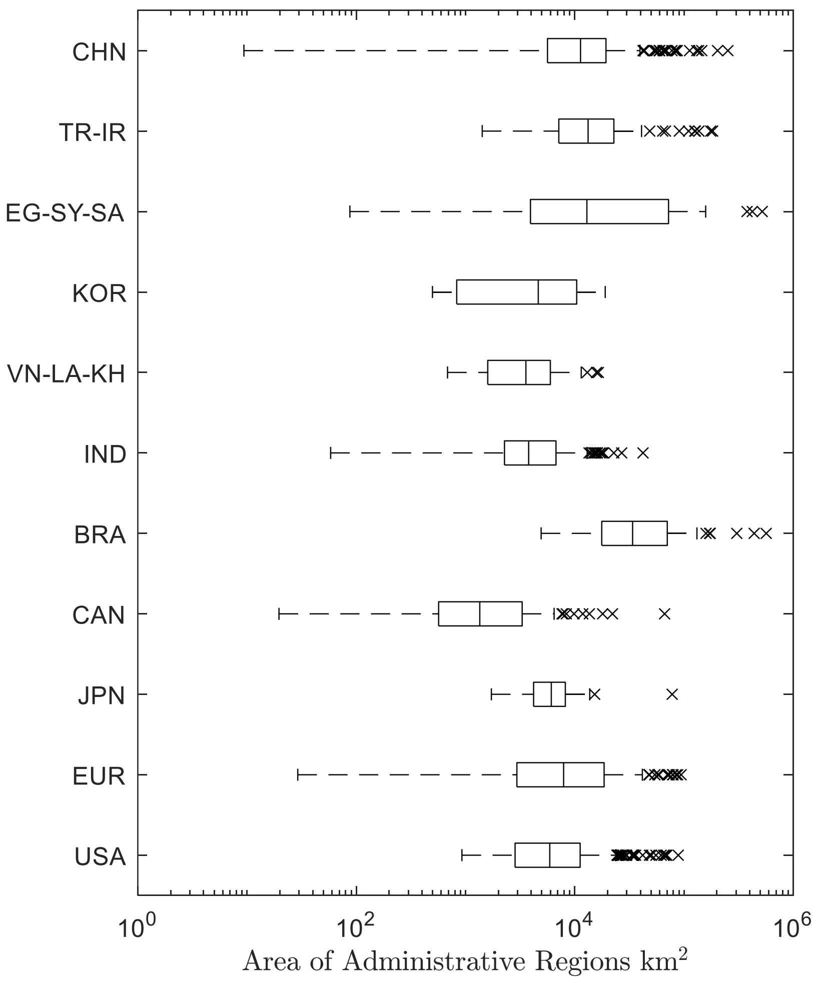

Figure S8. Land area of administrative regions in the economies included in this study.

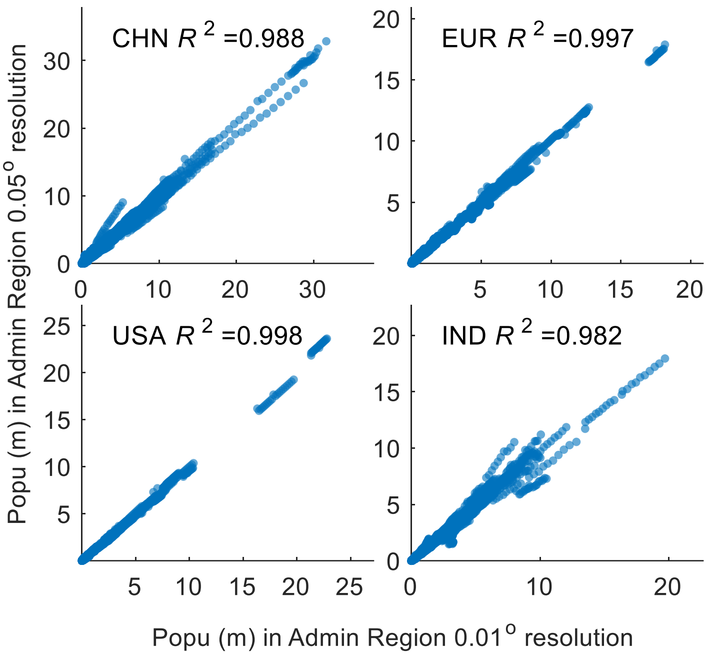

Figure S9. Comparison of city-level population estimates from 0.01° resolution geo-raster data against their upscaled counterparts at 0.05° resolution from 2000 to 2020.

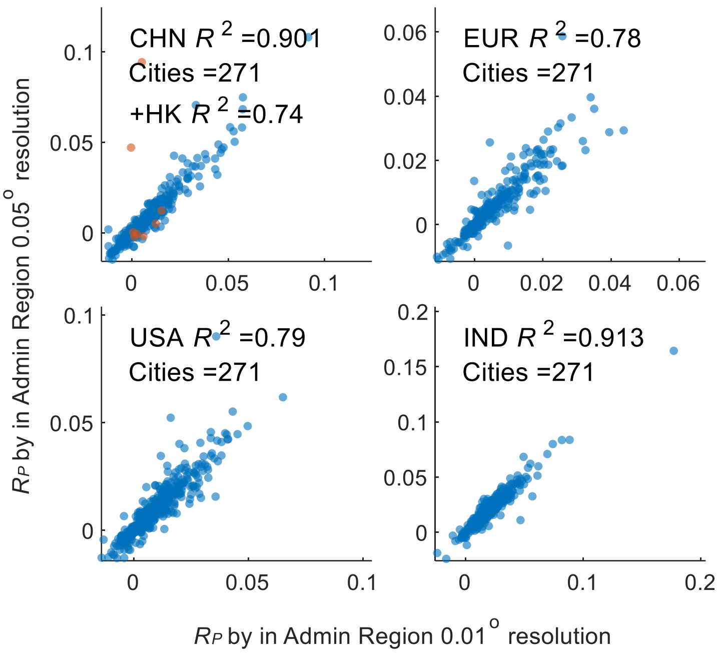

Figure S10. Comparison of city-level population growth rates estimated from 0.01° and upscaled 0.05° geo-raster resolutions from 2000 to 2020, with CHN denoting mainland China.

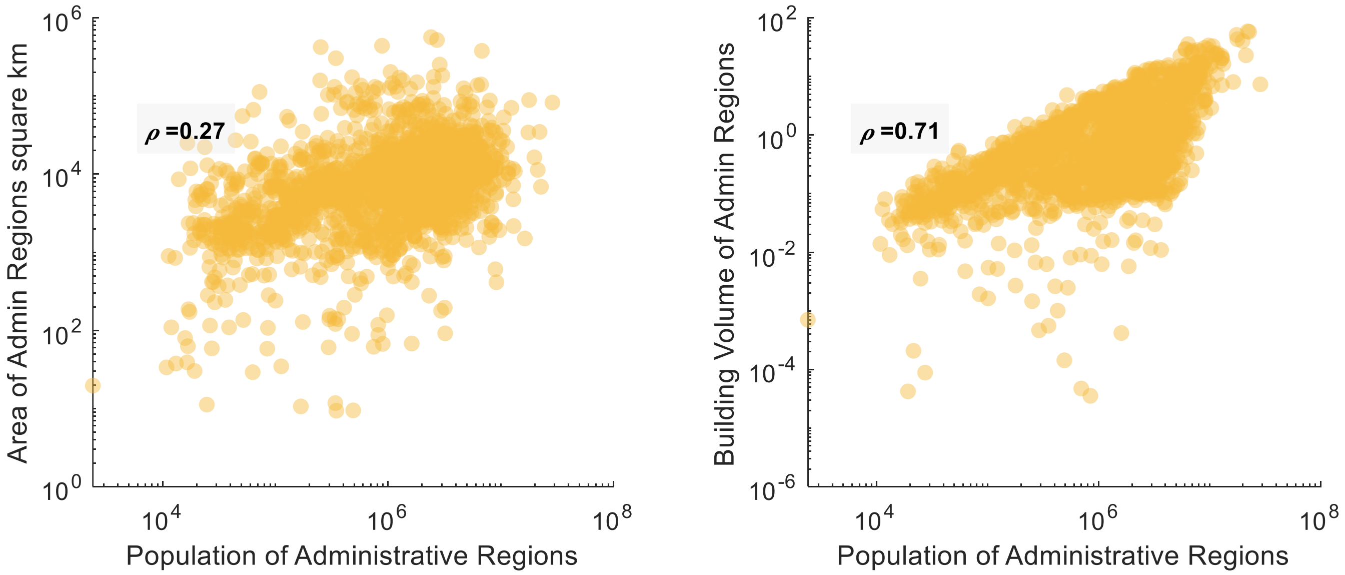

Figure S11. Correlations between administrative region population and land area (left), and building volume (right).

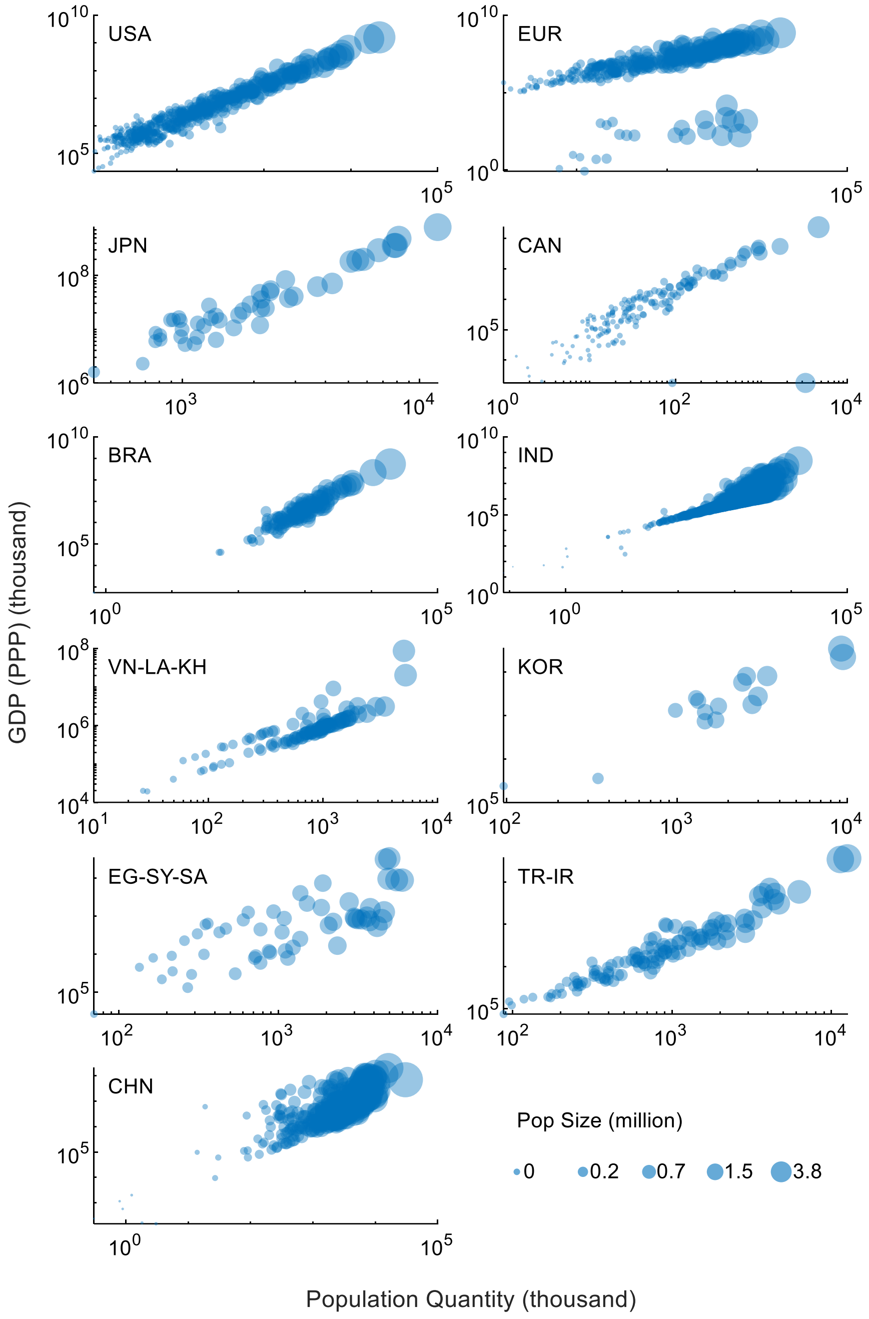

Figure S12. Correlation between population size and GDP (PPP-adjusted) across administrative regions worldwide.

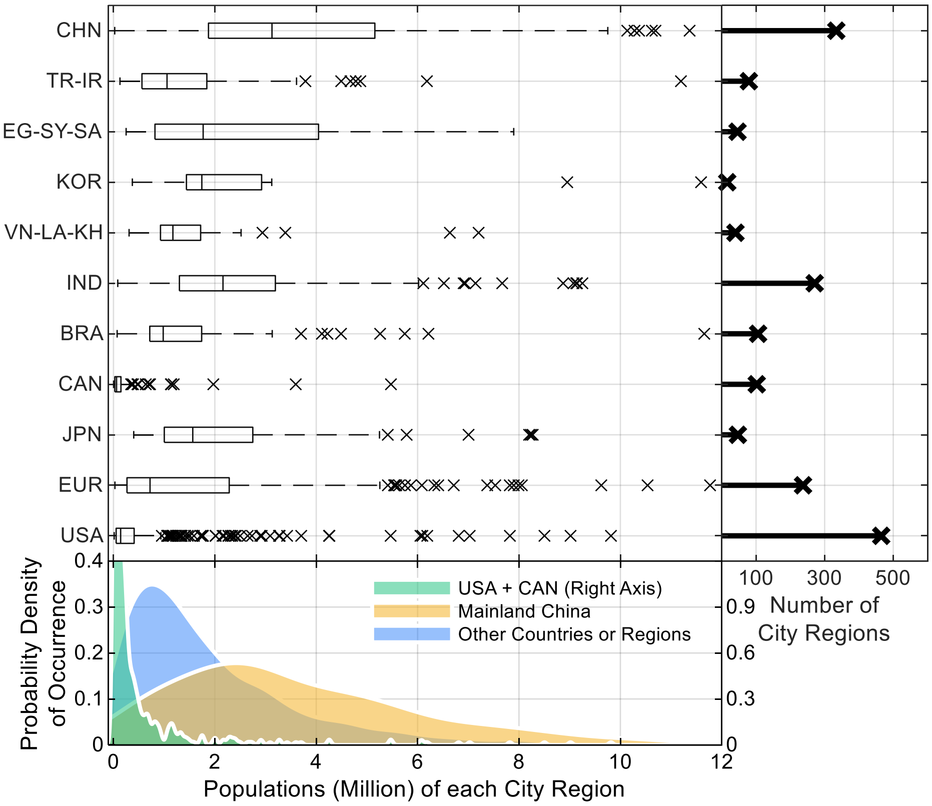

Figure S13. City population size across countries and regions, with occurrence probability densities of selected areas (China, North America, and others) shown by the curves.

Table S1. Administrative region levels for different countries and regions.

Countries	Abbre-viation	Place in the word	Administrative regions	Number of regions[a]
China	CHN	East Asia	Second-level (Prefecture-level city or region)	331
India	IND	South Asia	Second-level (Districts)	268
Japan	JPN	East Asia	First-level	47
South Korea	KOR	East Asia	First-level	16
Vietnam, Laos, Cambodia	VN-LA-KH	Southeast Asia	First-level	40
Brazil	BRZ	South America	RGI	98
United States	USA	North America	CSA and micropolitan statistical regions	429
Canada	CAN	North America	CMA and CA	97
Austria, Belgium, Czech, Denmark, Finland, France, Germany, Ireland, Italy, Luxembourg, Netherlands, Norway, Poland, Portugal, Spain, Sweden, Switzerland, United Kingdom[b]	EUR	Europe	First-level	229
Iran and Turkey	TR-IR	Middle East	First-level	74
Egypt, Saudi Arabia, Syria	EG-SY-SA	Middle East	First-level	44

[a]The number of regions with building volume higher than 0 and the coefficient of determination of the building volume and population's linear trend higher than 0. Please refer to supplementary notes and Figure S1 for details.

[b]The United Kingdom is sub-divided into 12 regions, following the former European Parliament constituencies in the United Kingdom
